# Mechanisms of tamoxifen resistance: insight from long non-coding RNAs

**DOI:** 10.3389/fonc.2024.1458588

**Published:** 2024-10-08

**Authors:** Yuxin Yan, Jian Zhang

**Affiliations:** ^1^ Department of Medical Oncology, Fudan University Shanghai Cancer Center, Shanghai, China; ^2^ Department of Oncology, Shanghai Medical College, Fudan University, Shanghai, China; ^3^ Phase I Clinical Trial Center, Fudan University Shanghai Cancer Center, Shanghai, China

**Keywords:** breast cancer, lncRNA, tamoxifen, drug resistance, mechanism

## Abstract

Breast cancer(BC) is the second most prevalent tumor in the world and one of the most lethal tumors in women. Patients with estrogen receptor-positive breast cancer can obtain significant advantages from endocrine therapies including tamoxifen, aromatase inhibitors, and others. However, the development of primary or acquired drug resistance ultimately leads to discontinuation of treatment with adverse consequences for breast cancer patients, and the underlying mechanisms have not been fully elucidated. Long non-coding RNAs (lncRNAs) play pivotal roles in orchestrating fundamental biochemical and cellular processes. They exert regulatory control over various processes including epigenetics, gene transcription, post-transcriptional modifications, and translation. Additionally, they influence key biological events like cell cycle progression, cell differentiation, and development. For the past few years, the relationship between lncRNAs and endocrine resistance has gained increasing attention, leading to a surge in related studies. LncRNAs mediate tamoxifen resistance in cancer by utilizing a variety of molecular mechanisms, including enhanced estrogen receptor (ER) signaling, inhibition of apoptosis, autophagy, exosome-mediated transfer, epigenetic alterations, epithelial-to-mesenchymal transition, and acting as competitive endogenous RNAs(ceRNAs). In this comprehensive review, we systematically summarize the critical role and intricate molecular mechanisms by which lncRNAs influence the development of tamoxifen resistance in breast cancer. Furthermore, we propose the potential clinical significance of lncRNAs as innovative therapeutic targets and prognostic biomarkers for breast cancer.

## 1 Introduction

Breast cancer (BC) holds the highest incidence rate among diagnosed malignancies in women and presents a great threat to female health worldwide (1). Approximately 70% of breast tumors exhibit estrogen receptor positive (ER+) (2) Consequently, specifically targeting the ER signaling pathway offers an efficacious approach for treating (ER+) breast cancer. Tamoxifen, known as selective estrogen receptor modulators (SERMs), is widely used in endocrine therapy. However, the development of tamoxifen resistance remains a daunting challenge during the treatment, which may lead to a poor prognosis. Tamoxifen resistance encompasses intricate mechanisms, involving somatic alterations, epigenetic modifications, and shifts in the tumor microenvironment (3).

Noncoding RNAs (ncRNAs) represent a burgeoning category of transcripts coded by the genome, yet predominantly lacking protein-coding functionality. ncRNAs are categorized as small non-coding RNAs (sncRNAs, 18~200 nucleotides) and long non-coding RNAs (lncRNAs, >200 nucleotides) according to their sizes (4). lncRNAs are involved in diverse functions, such as regulation of transcription, epigenetic modifications, protein/RNA stability, translation, and posttranslational modifications by interacting with DNA (5). In recent years, numerous investigations have demonstrated the dysregulation of lncRNAs in various cancers, influencing tamoxifen resistance through interactions with other RNAs and proteins associated with chemoresistance. These lncRNAs actively contribute to the intricate regulatory network in breast cancer by participating in various pathways and factors, ultimately influencing tamoxifen resistance (6). At present, an assortment of abnormally expressed lncRNAs are identified in breast cancer including DILA1, UCAT1, H19,

LINP1, SNHG6, CYTOR, HOTAIR (7), and other lncRNAs are summarized below.

In the current review, We conducted a comprehensive literature review to elucidate the intricate mechanisms by which lncRNAs either enhance or diminish tamoxifen resistance in breast cancer, thus suggesting lncRNAs promising biomarkers and therapeutic targets of breast cancer.

## 2 Classification of lncRNA in human breast cancers

**Figure 1:**
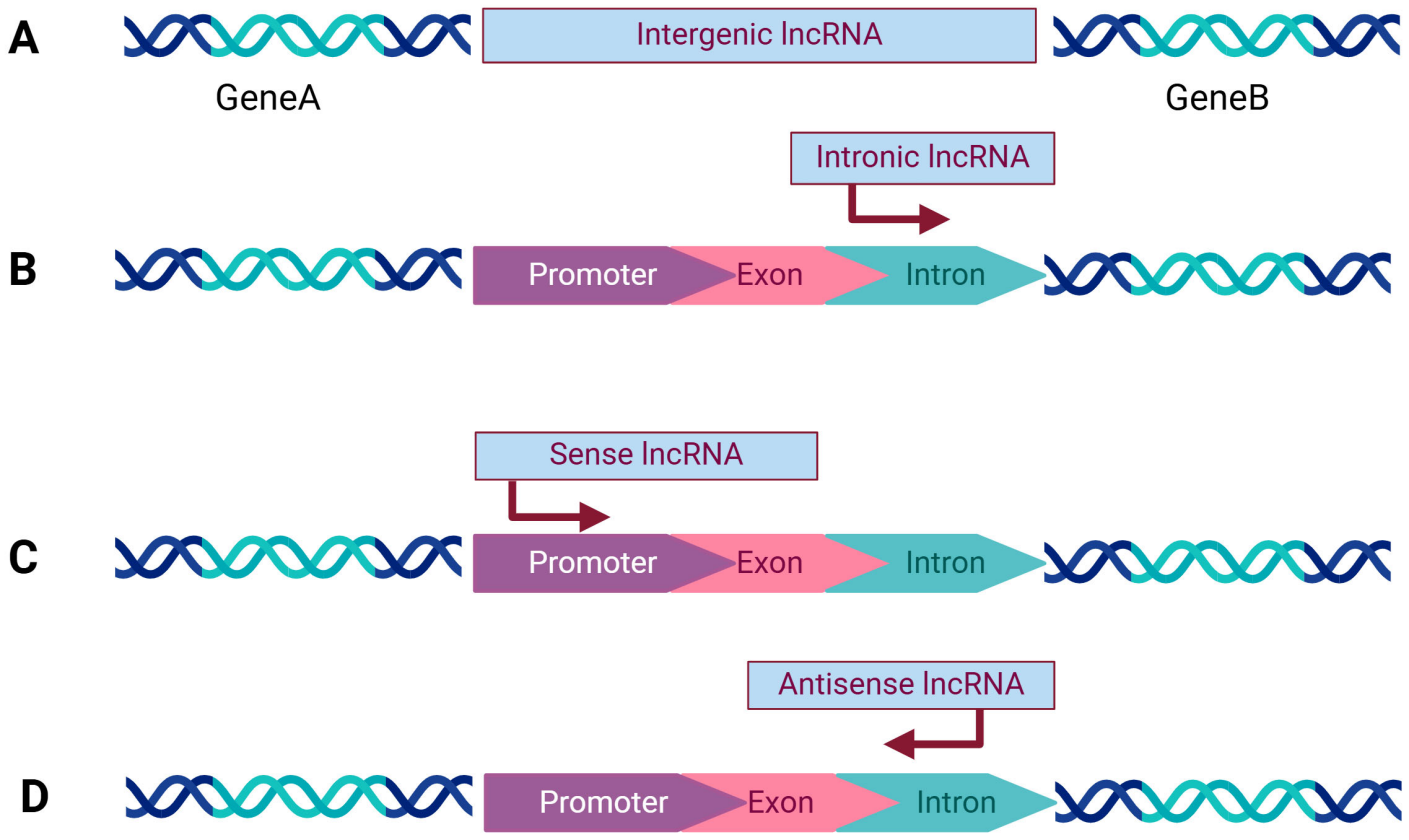
Categorization of lncRNAs: **(A)** Intergenic lncRNAs originate from the space between two protein-coding genes and are transcribed independently. **(B)** Intronic lncRNAs are composed of long introns transcribed from within the introns of other protein-coding genes. **(C)** Sense lncRNAs are are located within genes and share the same transcription orientation as the adjacent protein-coding gene. **(D)** Antisense lncRNAs are transcribed in the opposite direction to the corresponding protein-coding gene. Created with bioRender.com.

LncRNAs are categorized into numerous distinct groups to explore their underlying mechanisms of action (8) (**Figure 1**).

1. Intergenic lncRNAs (lincRNAs) are transcribed intergenically and play a crucial role in regulating gene expression through diverse mechanisms (9). While they can influence the expression of nearby genes, they can also regulate other genes by mechanisms such as miRNA sponging, modulation of transcriptional activity, and interaction with chromatin modifiers (10). lincRNAs undergo transcriptional activation most similar to mRNAs, demonstrating higher conservation and stability compared to intron transcripts.
2. Intronic lncRNAs, on the contrary, originate from intronic regions of protein-coding genes.
3. Sense lncRNAs are generated from the sense strand of protein-coding genes, incorporating exonic regions derived from protein-coding genes. These lncRNAs can partially overlap with protein-coding genes or encompass the entire sequence of a protein-coding gene.
4. Antisense lncRNAs, contrary to sense lncRNAs, originate from the antisense strand of protein-coding genes. Antisense transcripts may be in common use both in human and animal.

## 3 Functions of lncRNA

**Figure 2:**
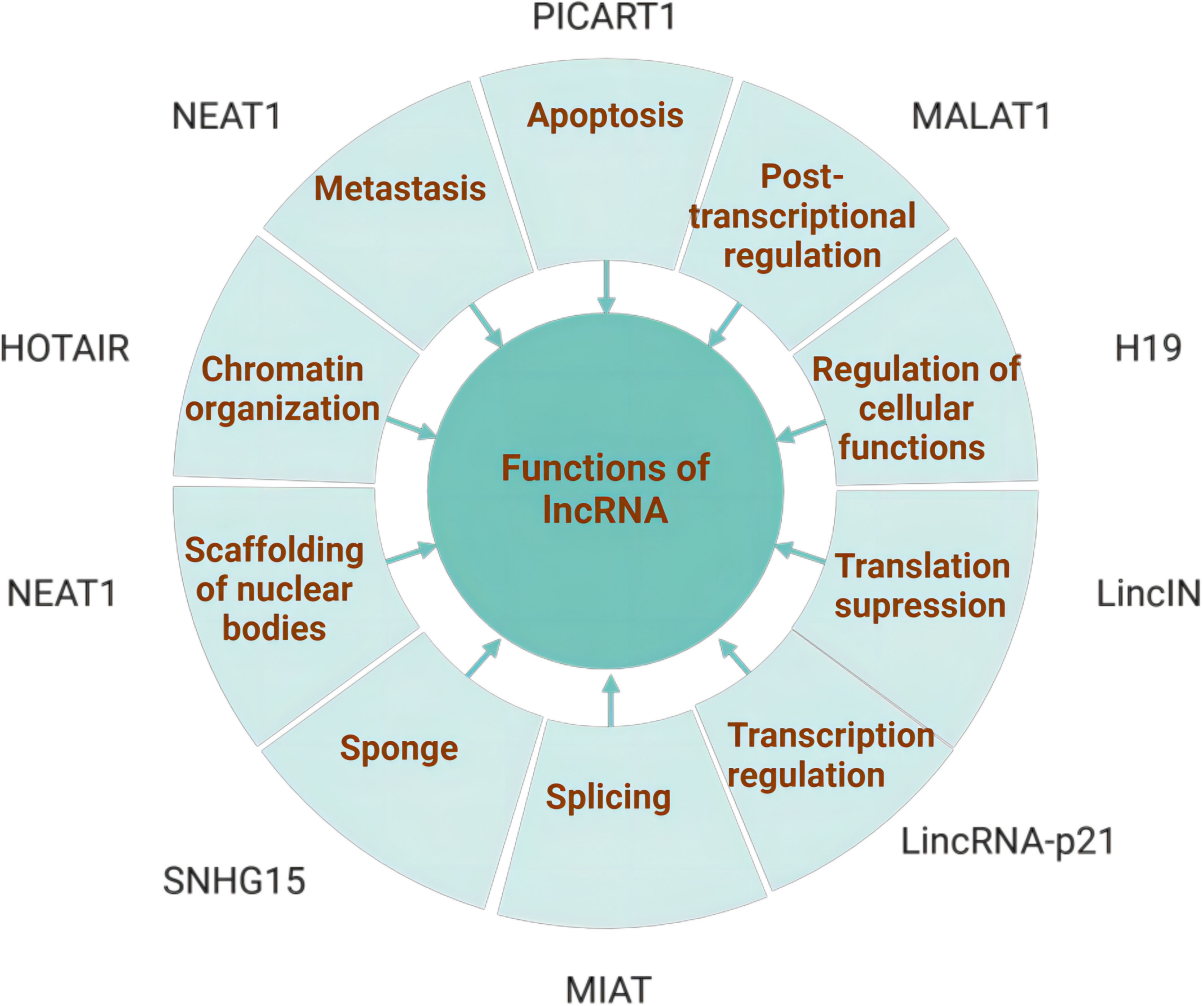
Functions of lncRNAs: The major functions of lncRNAs include chromatin organization, transcriptional regulation, sponging, scaffolding, apoptosis, metastasis, splicing modulation, post-transcriptional regulation, and regulation of cellular functions. A variety of lncRNAs involved in these functions are also associated with the tumorigenesis and progression. Created with bioRender.com.

LncRNAs have been proposed to present a variety of functions, including cis- or trans-transcriptional regulation, nuclear domain organization, and protein or RNA molecule regulation (11). (**Figure 2**) lncRNAs interacting with the mentioned molecules contribute to the coordination of various physiological processes, and their aberrant function are involved in a range of human diseases. Currently, they are recognized for their significant involvement in the regulation of cancer initiation, development and progression (12).

The functions of lncRNA are based on their unique subcellular localization. A large portion of lncRNAs are located in the nucleus (13) and participate in gene regulation at both the epigenetic and transcriptional levels (14), including histone modifications, alterations to chromatin structure, along with engagement with nuclear chromatin modification complexes, transcription factors and proteins. A handful of lncRNAs located in the cytoplasm engage in gene regulation at post-transcriptional and translational tiers. This involves interactions with cytoplasmic proteins and the modulation of mRNA metabolism, acting as ceRNAs that interact with microRNAs. To sum up, lncRNA plays a significant role in influencing cancer cell proliferation, migration, invasion, and resistance to therapeutic drugs.

For example, the lung cancer-related transcript 1 (LUCAT1) was first discovered in non-small cell lung cancer but highly expressed in various cancer (15). LUCAT1 is upregulated in breast cancer, including triple-negative breast cancer, where it promotes tumor progression and is linked to poor prognosis (16).Numerous studies have demonstrated that suppression of LUCAT1 expression can inhibit the advancement of breast cancer,indicating it as a novel therapy target. Mou et al. found that in TNBC tissues and cell lines, LUCAT1 expression is markedly elevated and strongly associated with unfavorable outcomes. Reducing LUCAT1 levels can suppress TNBC cell growth, movement, invasiveness, and epithelial-mesenchymal transition (EMT), while enhancing cell apoptosis. LUCAT1 operates through the competing endogenous RNA (ceRNA) mechanism, binding competitively to miR-5702 and consequently diminishing its expression.

## 4 LncRNA-mediated mechanisms of tamoxifen resistance

A great number of lncRNAs have been found to be abnormally expressed with the vast majority upregulated in breast cancer and involved in tamoxifen resistance through regulating different target genes. (**Table 1**) Most lncRNAs mediating tamoxifen resistance in BC play a facilitating role like LncRNA DILA1、H19、UCAT1 (17). This review uncovers several mechanisms through which lncRNAs influence tamoxifen resistance in breast cancer including enhanced ER signaling, suppression of apoptosis, autophagy, exosome-mediated transfer, epigenetic alterations, epithelial-to-mesenchymal transition and acting as ceRNA.

**Table 1:** LncRNAs contribute to tamoxifen resistance in breast cancer.

LncRNAs	Expression	Targeting Genes or Pathways	Effect on Resistance	Reference
DILA1	↑	GSK3β	Promoting	(17)
Lnc-DC	↑	STAT3	Promoting	(18)
UCAT1	↑	miR-7-5p,SOX2,miR-5702	Promoting	(16)
H19	↑	Beclin1	Promoting	(19)
LINP1	↑	ER signaling	Promoting	(20)
DSCAM-AS1	↑	miR-137	Promoting	(21)
MIR497HG	↓	miR-195、miR-497 PI3K/AKT	Reversing	(22)
ADAMTS9-AS2	↓	miR-130a-5p	Promoting	(23)
SNHG6	↑	miR-101	Promoting	(24)
ATXN8OS	↑	miR-16-5p	Promoting	(25)
CYTOR	↑	miR−125a−5p	Promoting	(26)
LncRNA-42060	↑	miR-204-5p/SOX4	Promoting	(27)
SBF2-AS1	↑	PI3K/AKT/MTOR	Promoting	(28)
HOTAIR	↑	ER signaling	Promoting	(29)
TUG-1	↑	miR-186	Promoting	(30)
MAFG-AS1	↑	miR-339-5p	Promoting	(31)
ELOVL2-AS1	↓	miR-1233-3p	Promoting	(32)
AGPG	↑	E2F1 signaling	Promoting	(33)
BNAT1	↑	ER signaling	Promoting	(34)
BDNF-AS	↑	mTOR signaling	Promoting	(35)
HNF1A-AS1	↑	miR-363/SERTAD3	Promoting	(36)
CCAT2	↑	ERK/MAPK signaling	Promoting	(37)
LINC00894-002	↑	TGF-β Signaling	Promoting	(38)
ROB	↑	Beclin-1 and light chain 3 (LC3)	Promoting	(39)

lncRNAs up-regulated (↑) in tamoxifen-resistant breast cancer cells.

lncRNAs down-regulated (↓) in tamoxifen-resistant breast cancer cells.

This table shows 24 lncRNAs whose expression levels and underlying pathways in tamoxifen resistance to breast cancer.

### 4.1 LncRNA and enhanced ER signaling

Estrogen receptor(ER) plays a critical role in breast cancer, acting as a master transcriptional regulator that shapes the phenotype of breast cancer and serves as a central target for molecular therapy (40). Targeting of proteins and genes within ER nuclear and nonnuclear pathways has generated a range of endocrine therapies. SERMs operate by “occupying” estrogen receptors in breast cells so that estrogen cannot bind to the receptors in breast cells and the cells do not receive estrogen signals to grow and reproduce. Tamoxifen, a type of SERM, is the cornerstone of endocrine therapy for ER(+) breast cancer but the development of drug resistance remains a challenging obstacle.

Recent studies have revealed that lncRNA HOTAIR is upregulated in tamoxifen-resistant breast cancer cells compared to primary breast cancer cells, which contributes to enhanced ER signaling, even in the absence of estrogen (29). HOTAIR enhances tamoxifen resistance through regulating ER at the level of transcription. Furthermore, the research indicates that reducing HOTAIR levels in tamoxifen-resistant MCF7 breast cancer cells markedly inhibits their growth. This finding implies that reversing tamoxifen resistance might be achievable by targeting and diminishing HOTAIR.

### 4.2 LncRNA and suppressed apoptosis

Apoptosis, often referred to as programmed cell death, is a highly regulated and controlled process that occurs naturally in the body. It plays a crucial role in maintaining the balance and health of tissues by eliminating senescent, damaged, or unnecessary cells. Cancer is one of the outcomes where too little apoptosis occurs, resulting in abnormal proliferation of cancer cells (41).Thus, de-regulated apoptotic signaling, including the intrinsic mitochondrial pathway and the extrinsic death receptor pathway, leads to drug resistance and recurrence of cancer (42). Long non-coding RNAs safeguard cancer cells by suppressing apoptosis triggered by oxidative stress or DNA damage caused by tamoxifen. For example, LncRNA PRNCR1 promotes breast cancer proliferation and inhibits apoptosis by modulating microRNA-377/CCND2/MEK/MAPK Axis (43). PRNCR1 inhibits miR-377, leading to the upregulation of CyclinD2 (CCND2), which is a distinct gene among the three D-type cyclins. This upregulation subsequently activates the MEK/MAPK pathway, resulting in the suppression of apoptosis and the regulation of proliferation in breast cancer cells.

### 4.3 LncRNA and autophagy

Autophagy is a cellular process that involves the degradation and recycling of cellular components, such as damaged organelles and misfolded proteins, to maintain cellular homeostasis and survival. Mutations in autophagy-related processes are associated with various physiological and pathological conditions, including stress response, aging, infection, and cancer (44). In multiple settings, autophagy is regarded as a double-edged sword in tumor therapy (45). In healthy cells, autophagy typically prevents the transformation into cancer cells. However, in cancer cells, efficient autophagic responses can aid in tumor progression and resistance to treatments. LncRNAs may interact with key targets to influence the resistance of breast cancer cells to tamoxifen during the autophagy process​.

Li Y et al. explored the mechanism underlying the lncRNA regulator of reprogramming (ROR) modulating autophagy on tamoxifen resistance in breast cancer. They demonstrated that lncRNA ROR expression in the BC tissues was five times higher than in the paraneoplastic tissues.

*In vitro* studies have demonstrated that silencing ROR through small interfering RNA can enhance autophagy and elevate tamoxifen sensitivity by elevating the levels of two key autophagic markers—Beclin-1 and microtubule-associated protein 1A/1B-light chain 3 (LC3) (39).On the contrary, Wang J et al. found that lncRNA H19 promotes tamoxifen resistance in breast cancer via autophagy as well. *In vitro* cell culture revealed that the expression of H19 in tamoxifen-resistant MCF-7 BCE cells and tumor tissues was upregulated. Downregulation of H19 could inhibit autophagy and reverse resistance to tamoxifen (19).

In the case of tamoxifen, the response differs significantly, primarily due to the diverse types of BC cells. While the intricate molecular interplay between lncRNAs and autophagy is not fully understood, it is clear that various lncRNAs influence autophagy by regulating crucial autophagy-related genes, resulting in distinct outcomes in tamoxifen-resistant BC. A deeper comprehension of the multifaceted role of autophagy in tamoxifen resistance could pave the way for innovative strategies to overcome this resistance and identify new therapeutic targets for effective management.

### 4.4 Exosome-mediated transfer of lncRNA

Exosomes, which are a subset of extracellular vesicles (EVs), contain nucleic acids, amino acids, proteins, and lipids, with lncRNAs being one of the major components of their cargo (46). Cancer cells typically release more exosomes compared to healthy cells, and these cancer-derived exosomes possess a potent ability to alter microenvironments both locally and distantly (47). Furthermore, EVs play significant roles in mediating drug resistance through various mechanisms. In recent studies, lncRNAs associated with exosomes have been identified as key factors in promoting drug resistance in BC.C-G Xu et al. (48) compared the loading of Urothelial carcinoma-associated 1(UCA1) in exosomes released from tamoxifen-sensitive MCF-7 cells and tamoxifen-resistant LCC2 cells and found that UCA1 is significantly increased in exosomes from tamoxifen-resistant cells than that from tamoxifen sensitive cells. Furthermore, mTOR signaling is hypothesized to be an important downstream signaling pathway of UCA1 in tamoxifen resistance which requires further studies.

### 4.5 Epigenetic modifications of LncRNAs

Building upon the foundation of genomic and epigenomic mechanisms, lncRNAs introduce an extra layer of regulation. They facilitate both transcriptional and post-transcriptional control by engaging with proteins and nucleic acids that modulate gene expression within the nucleus and cytoplasm. Numerous lncRNAs, including HOTAIR, ANRIL, ROR, and H19, play a role in suppressing transcription through the recruitment of proteins involved in chromatin remodeling or histone modification.

Cyclin D1 is a key oncoprotein that promotes the proliferation of cancer cells and is linked to tamoxifen resistance in breast cancer. The lncRNA DILA1, which interacts with Cyclin D1, is found to be upregulated in tamoxifen-resistant breast cancer cells (17). Mechanistically, DILA1 prevents the phosphorylation of Cyclin D1 at the Thr286 site by directly binding to it, thereby inhibiting its degradation. This leads to an increased level of Cyclin D1 protein in breast cancer cells. Yu et al. (33) investigated that in endocrine-resistant breast cancer cells, the expression of the lncRNA actin gamma 1 pseudogene 25 (AGPG) was found to be elevated, a phenomenon attributed to the epigenomic activation of an enhancer. AGPG engaged in a physical interaction with PURα, which in turn freed E2F1 from PURα’s grasp, thereby triggering the activation of E2F1 signaling pathways in ERα-positive breast cancer cells.

### 4.6 LncRNA induced EMT

The epithelial-to-mesenchymal transition (EMT) is a cellular program crucial for diverse pathological events, such as wound repair, tissue scarring, and the advancement of cancer (49). An increasing number of research has revealed the activation of EMT throughout the Development of malignant tumors (50). Khan and Ahmad (24) found that the expression of SNHG6 is elevated in tamoxifen-resistant cells and positively regulates acquired resistance against tamoxifen. It sponges miR-101, leading to the activation of EMT and increased EMT cell markers including E-cadherin, vimentin, ZEB1 and ZEB2, contributing to tamoxifen resistance. Silencing SNHG6 sensitizes resistant cells to tamoxifen, reverses EMT, and reduces invasion and clonogenicity, implicating the SNHG6-miR-101 axis as a potential target for overcoming tamoxifen resistance in breast cancer.

### 4.7 lncRNAs act as competitive endogenous RNA

MicroRNAs (miRNAs) are sncRNAs that play important roles in posttranscriptional gene regulation. They act by inhibiting translation and decreasing the stability of target RNAs (30). Salmena et al. hypothesized that lncRNAs can function as competitive endogenous RNA(ceRNA) to bind specific miRNAs, which in turn could alleviate the repression of their target mRNAs. Additionally, microRNAs can also be “sponged” or titrated away by lncRNAs, thereby exerting their effects independently of protein coding.

In addition to the inducing of EMT mentioned above, SNHG6 was also found to sponge and inhibit miR-101, with the endogenous expression levels of SNHG6 and miR-101 being inversely correlated. Moreover, silencing SNHG6 in tamoxifen-resistant cells resulted in miR-101 inhibition and reversed EMT. SNHG6 directly correlated with increased stem cell markers Sox2, Oct4, and EZH2. Manipulation of miR-101 levels affected tamoxifen sensitivity, with pre-miR-101 sensitizing resistant cells to tamoxifen and anti-miR-101 inducing resistance in parental cells. MiR-101 was found to attenuate SNHG6-mediated effects on tamoxifen resistance, EMT, and stem cell markers, presenting a crucial role for the SNHG6-miR-101 axis in tamoxifen resistance of ER-positive breast cancer cells.

Chen X et al. (51) found that LINC02568 regulates estrogen receptor-induced transcriptional activation of target genes in the cytoplasm by competitively binding miR-1233-5p to the ESR1 mRNA of estrogen receptor, thereby trans-regulating ESR1 mRNA stability. Meanwhile, LINC02568 plays a specific role in maintaining the pH homeostasis of tumors by controlling the activity of the carbonic anhydrase enzyme CA12 in the nucleus through a cis-regulatory mechanism. Targeting the antisense nucleotide (ASO) of LINC02568 significantly inhibited the growth as well as tumor formation of estrogen receptor-positive breast cancer cells and restored the sensitivity of tamoxifen-resistant breast cancer cells to tamoxifen.

## 5 Cancer therapy for targeting lncRNAs

Tamoxifen, prescribed as a gold standard treatment for ER-positive BC, has been shown to substantially reduce the recurrence rate by 40% and the mortality rate by 30%. However, even with 5 years of tamoxifen treatment, one-third of these patients experience a relapse within 15 years (52). Endocrine-resistant patients, who may constitute up to 25% of all breast cancer cases, continue to pose a significant clinical challenge. Extensive efforts have been pursued to treat tamoxifen-resistant patients. At ESMO 2022, third-generation oral SERM lasofoxifene shined. Phase II ELAINE 1 study results show lasofoxifene delivers durable complete remission (CR) in ER-positive, HER-negative, metastatic breast cancer patients harboring ESR1 mutation (53).

In this context, lncRNAs emerge as promising therapeutic targets due to their involvement in regulating oncogenic molecular networks and their cell type-specific expression. They can act as oncogenes or tumor suppressors, influencing various cancer hallmarks such as cell proliferation, invasion, and metastasis. Despite their potential, targeting lncRNAs faces several hurdles including their structural complexity, diversity in function, and poor annotation.

Currently various pharmaceutical strategies targeting lncRNAs aim to achieve loss-of-function (LOF) in cancer therapy (54). LOF involves the use of oligonucleotides such as antisense oligonucleotides (ASOs) and small interfering RNAs (siRNAs) to downregulate harmful lncRNAs. Gapmer ASOs bind to target lncRNAs based on sequence complementarity. Once bound, they induce degradation of the lncRNA by recruiting RNase H1 in the nucleus. This approach is effective regardless of the lncRNA’s subcellular localization (55). SiRNAs are double-stranded RNA molecules that also target lncRNAs through sequence complementarity. The active strand of the siRNA is incorporated into the RNA-induced silencing complex (RISC), which then binds to and cleaves the complementary lncRNA, leading to its degradation. SiRNAs mainly function in the cytoplasm, though ongoing debate exists about their activity in the nucleus. Several oncogenic lncRNAs have been successfully targeted using siRNAs, including SNHG6 (24), ELOVL2-AS1 (32), BNAT1 (34).

Zhimin Shao et al. conducted a clinical trial (NCT02641847) aimed at evaluating the efficacy and safety of two chemotherapy regimens in high-risk triple-negative breast cancer (TNBC) patients, as identified by an integrated mRNA-lncRNA signature. The trial aims to validate this signature’s ability to predict recurrence risk in TNBC patients and explore optimal chemotherapy strategies. Patients classified as high-risk are randomized to receive either a regimen of docetaxel, doxorubicin (or epirubicin), and cyclophosphamide followed by gemcitabine and cisplatin, or doxorubicin (or epirubicin) and cyclophosphamide followed by docetaxel. The primary goal is to assess recurrence-free survival, with secondary endpoints including safety and overall survival.

An ongoing clinical trial (NCT06307249) has been launched to explore the therapeutic potential of combining Palbociclib, a CDK4/6 inhibitor, with Bevacizumab, a VEGF inhibitor, in treating solid tumors, with an initial focus on colorectal cancer and plans to extend to other cancers, including breast cancer. The study is particularly innovative in its incorporation of LncRNAs as biomarkers, which have shown promise in predicting treatment response and prognosis across various cancers. By analyzing the molecular profiles through LncRNA markers, the trial aims to enhance the efficacy of this combination therapy, offering a targeted and individualized treatment option for patients with solid tumors, ultimately striving for better outcomes and improved survival rates.

Advanced genomic technologies and molecular tools are used to identify and validate lncRNA targets. These include high-throughput RNA sequencing, CRISPR-Cas technologies for genomic editing, and various *in vivo* and *in vitro* models for functional validation (56).

## 6 Conclusion

As master gene regulators, lncRNAs have been implicated in the regulation of diverse cellular functions and disease processes. Substantial knowledge on how lncRNAs contribute to cancer has accumulated in the last 10 years and extensive efforts have been fostered to discover mechanisms of drug resistance and potential targets for the treatment of breast cancer patients. ER is not only a good diagnostic marker for breast cancer but also serves as a therapeutic target. Tamoxifen is one of the important SERMs for ER(+)breast cancers and it competitively inhibits the recruitment of transcription coactivators by ER, hence shutting down the transcription of ER-responsive genes. However, a critical challenge is the development of tamoxifen resistance.

**Figure 3:**
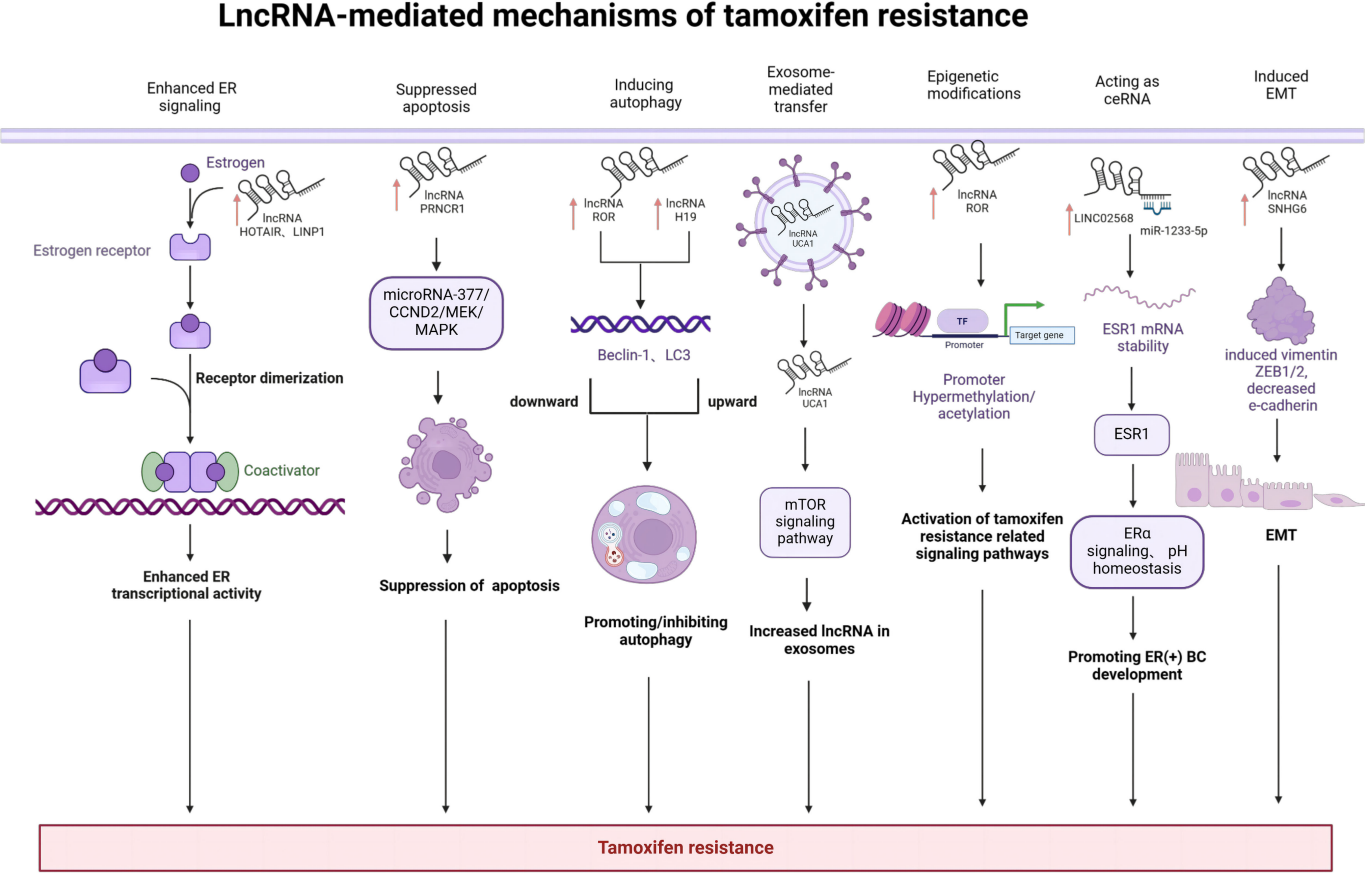
LncRNA-mediated mechanisms of tamoxifen resistance in breast cancer: LncRNAs mediate drug resistance by various molecular mechanisms including enhanced ER signaling, suppression of apoptosis, autophagy, exosome-mediated transfer, epigenetic alterations, epithelial-to-mesenchymal transition and acting as ceRNA. Created with bioRender.com.

Mechanisms of tamoxifen resistance are complex and may involve multiple factors such as AKT, HER2, and R-Ras. These studies have been focusing on protein-coding genes. On the other hand, much less is known about the role of lncRNAs in tamoxifen resistance although the number of lncRNAs is much larger than that of protein-coding genes. Thus, our study provides new insight into the lncRNA-mediated mechanisms of tamoxifen resistance in BC including enhanced ER signaling, suppression of apoptosis, autophagy, exosome-mediated transfer, epigenetic alterations, epithelial-to-mesenchymal transition and acting as ceRNA. A comprehensive mechanism related to recently identified various novel lncRNA implicated in BC tamoxifen resistance has been summarized. (**Figure 3**) Combination of lncRNAs-based therapeutic interventions with traditional chemotherapy or targeted therapy may be a promising option to conquer tamoxifen resistance in ER(+)breast cancer patients. However, it is still a challenge how to choose crucial target lncRNAs from a large amount of candidate lncRNAs. Future translational studies or clinical trials are warranted to develop lncRNAs-based therapeutics, which may eventually improve the prognosis of tamoxifen-resistant breast cancer patients.

## References

[1] Harbeck N Gnant M. Breast cancer, lancet lond. Engl. (2017) 389:1134–50. doi: 10.1016/S0140-6736(16)31891-8 27865536

[2] DeSantis CE Ma J Gaudet MM Newman LA Miller KD Goding Sauer A. Breast cancer statistics, 2019, CA. Cancer J Clin. (2019) 69:438–51. doi: 10.3322/caac.21583 31577379

[3] Overcoming endocrine resistance in breast cancer (n.). Available online at: https://pubmed.ncbi.nlm.nih.gov/32289273/ (Accessed November 12, 2023).

[4] Esteller M Pandolfi PP. The epitranscriptome of noncoding RNAs in cancer. Cancer Discov. (2017) 7:359–68. doi: 10.1158/2159-8290.CD-16-1292 PMC5997407 28320778

[5] Bridges MC Daulagala AC Kourtidis A. LNCcation: LncRNA localization and function, J. Cell Biol. (2021) 220:e202009045. doi: 10.1083/jcb.202009045 PMC7816648 33464299

[6] Amelio I Bernassola F Candi E. Emerging roles of long non-coding RNAs in breast cancer biology and management, Semin. Cancer Biol. (2021) 72:36–45. doi: 10.1016/j.semcancer.2020.06.019 32619506

[7] Su X Malouf GG Chen Y Zhang J Yao H Valero V. Comprehensive analysis of long non-coding RNAs in human breast cancer clinical subtypes. Oncotarget. (2014) 5:9864–76. doi: 10.18632/oncotarget.2454 PMC4259443 25296969

[8] Ma L Bajic VB Zhang Z. On the classification of long non-coding RNAs. RNA Biol. (2013) 10:925–33. doi: 10.4161/rna.24604 PMC4111732 23696037

[9] The functions and unique features of long intergenic non-coding RNA . Available online at: https://pubmed.ncbi.nlm.nih.gov/29138516/ (Accessed November 13, 2023).

[10] Tang S-S Zheng B-Y Xiong X-D. LincRNA-p21: Implications in human diseases, Int. J Mol Sci. (2015) 16:18732–40. doi: 10.3390/ijms160818732 PMC4581268 26270659

[11] Functional classification and experimental dissection of long noncoding RNAs (n.). Available online at: https://pubmed.ncbi.nlm.nih.gov/29373828/ (Accessed November 21, 2023). 10.1016/j.cell.2018.01.011 PMC5978744 29373828

[12] Yang M Lu H Liu J Wu S Kim P Zhou X. LncRNAfunc: A knowledgebase of lncRNA function in human cancer. Nucleic Acids Res. (2022) 50:D1295–D1306. doi: 10.1093/nar/gkab1035 34791419 PMC8728133

[13] Localization and abundance analysis of human lncRNAs at single-cell and single-molecule resolution (n.). Available online at: https://pubmed.ncbi.nlm.nih.gov/25630241/ (Accessed November 22, 2023). 10.1186/s13059-015-0586-4 PMC4369099 25630241

[14] Genome-wide analysis of long noncoding RNA stability (n.). Available online at: https://genome.cshlp.org/content/22/5/885 (Accessed November 22, 2023).

[15] Xing C Sun SG Yue ZQ Bai F. Role of lncRNA LUCAT1 in cancer. Biomed Pharmacother. (2021) 134:111158. doi: 10.1016/j.biopha.2020.111158 33360049

[16] LncRNA LUCAT1 facilitates tumorigenesis and metastasis of triple-negative breast cancer through modulating miR-5702 (n.). Available online at: https://pubmed.ncbi.nlm.nih.gov/31399501/ (Accessed November 30, 2023). 10.1042/BSR20190489 PMC6722493 31399501

[17] Shi Q Li Y Li S Jin L Lai H Wu Y. LncRNA DILA1 inhibits cyclin d1 degradation and contributes to tamoxifen resistance in breast cancer, Nat. Commun. (2020) 11:5513. doi: 10.1038/s41467-020-19349-w PMC7608661 33139730

[18] Peng W-X Koirala P Zhou H Jiang J Zhang Z Yang L. Lnc-DC promotes estrogen independent growth and tamoxifen resistance in breast cancer. Cell Death Dis. (2021) 12:1000. doi: 10.1038/s41419-021-04288-1 34697301 PMC8546148

[19] The long noncoding RNA h19 promotes tamoxifen resistance in breast cancer via autophagy (n.). Available online at: https://pubmed.ncbi.nlm.nih.gov/31340867/ (Accessed October 23, 2023). 10.1186/s13045-019-0747-0 PMC6657081 31340867

[20] Ma T Liang Y Li Y Song X Zhang N Li X. LncRNA LINP1 confers tamoxifen resistance and negatively regulated by ER signaling in breast cancer, Cell. Signal. (2020) 68:109536. doi: 10.1016/j.cellsig.2020.109536 31927036

[21] Ma Y Bu D Long J Chai W Dong J. LncRNA DSCAM-AS1 acts as a sponge of miR-137 to enhance tamoxifen resistance in breast cancer, J. Cell Physiol. (2019) 234:2880–94. doi: 10.1002/jcp.27105 30203615

[22] Tian Y Chen Z-H Wu P Zhang D Ma Y Liu X-F. MIR497HG-derived miR-195 and miR-497 mediate tamoxifen resistance via PI3K/AKT signaling in breast cancer, Adv. Sci Weinh. Baden-Wurtt. Ger. (2023) 10:e2204819. doi: 10.1002/advs.202204819 PMC10131819 36815359

[23] Shi Y-F Lu H Wang H-B. Downregulated lncRNA ADAMTS9-AS2 in breast cancer enhances tamoxifen resistance by activating microRNA-130a-5p, Eur. Rev Med Pharmacol Sci. (2019) 23:1563–73. doi: 10.26355/eurrev_201902_17115 30840279

[24] Khan MI Ahmad A. LncRNA SNHG6 sponges miR-101 and induces tamoxifen resistance in breast cancer cells through induction of EMT, Front. Oncol. (2022) 12:1015428. doi: 10.3389/fonc.2022.1015428 PMC9539827 36212408

[25] Zhang H Zhang J Dong L Ma R. LncRNA ATXN8OS enhances tamoxifen resistance in breast cancer, Open Med. Wars. Pol. (2021) 16:68–80. doi: 10.1515/med-2021-0012 PMC7754175 33385064

[26] LncRNA CYTOR promotes tamoxifen resistance in breast cancer cells via sponging miR−125a−5p (n.). Available online at: https://pubmed.ncbi.nlm.nih.gov/31894257/ (Accessed November 30, 2023).

[27] Xu E Hu M Ge R Tong D Fan Y Ren X. LncRNA-42060 regulates tamoxifen sensitivity and tumor development via regulating the miR-204-5p/SOX4 axis in canine mammary gland tumor cells, Front. Vet Sci. (2021) 8:654694. doi: 10.3389/fvets.2021.654694 PMC8255626 34235197

[28] Hussain SA Venkatesh T. YBX1/lncRNA SBF2-AS1 interaction regulates proliferation and tamoxifen sensitivity via PI3K/AKT/MTOR signaling in breast cancer cells, Mol. Biol Rep. (2023) 50:3413–28. doi: 10.1007/s11033-023-08308-5 36754932

[29] Xue X Yang YA Zhang A Fong K-W Kim J Song B. LncRNA HOTAIR enhances ER signaling and confers tamoxifen resistance in breast cancer. Oncogene. (2016) 35:2746–55. doi: 10.1038/onc.2015.340 PMC4791209 26364613

[30] Azzam HN El-Derany MO Wahdan SA Faheim RM Helal GK El-Demerdash E. Metabolic/hypoxial axis predicts tamoxifen resistance in breast cancer, Sci. Rep. (2022) 12:16118. doi: 10.1038/s41598-022-19977-w PMC9515205 36167713

[31] Feng J Wen T Li Z Feng L Zhou L Yang Z. Cross-talk between the ER pathway and the lncRNA MAFG-AS1/miR-339-5p/CDK2 axis promotes progression of ER+ breast cancer and confers tamoxifen resistance. Aging. (2020) 12:20658–83. doi: 10.18632/aging.103966 PMC7655217 33098638

[32] Kim HW Baek M Jung S Jang S Lee H Yang S-H. ELOVL2-AS1 suppresses tamoxifen resistance by sponging miR-1233-3p in breast cancer. Epigenetics. (2023) 18:2276384. doi: 10.1080/15592294.2023.2276384 37908128 PMC10621244

[33] Yu S Wang Y Gong X Fan Z Wang Z Liang Z. LncRNA AGPG confers endocrine resistance in breast cancer by promoting E2F1 activity. Cancer Res. (2023) 83:3220–36. doi: 10.1158/0008-5472.CAN-23-0015 37463119

[34] Horie K Takagi K Takeiwa T Mitobe Y Kawabata H Suzuki T. Estrogen-inducible LncRNA BNAT1 functions as a modulator for estrogen receptor signaling in endocrine-resistant breast cancer cells. Cells. (2022) 11:3610. doi: 10.3390/cells11223610 36429038 PMC9688125

[35] Lin X Dinglin X Cao S Zheng S Wu C Chen W. Enhancer-driven lncRNA BDNF-AS induces endocrine resistance and Malignant progression of breast cancer through the RNH1/TRIM21/mTOR cascade. Cell Rep. (2020) 31:107753. doi: 10.1016/j.celrep.2020.107753 32521278

[36] Li Y Liu L Lv Y Zhang Y Zhang L Yu H. Silencing long non-coding RNA HNF1A-AS1 inhibits growth and resistance to TAM of breast cancer cells via the microRNA-363/SERTAD3 axis, J. Drug Target. (2021) 29:742–53. doi: 10.1080/1061186X.2021.1878362 33472456

[37] Caia Y He J Zhang D. Suppression of long non-coding RNA CCAT2 improves tamoxifen-resistant breast cancer cells’ response to tamoxifen. Mol Biol. (2016) 50:725–30. doi: 10.1134/S0026893316030043 27830684

[38] Zhang X Wang M Sun H Zhu T Wang X. Downregulation of LINC00894-002 contributes to tamoxifen resistance by enhancing the TGF-β signaling pathway. Biochem Mosc. (2018) 83:603–11. doi: 10.1134/S0006297918050139 29738694

[39] Li Y Jiang B Zhu H Qu X Zhao L Tan Y. Inhibition of long non-coding RNA ROR reverses resistance to tamoxifen by inducing autophagy in breast cancer, Tumour Biol. J Int Soc Oncodevelopmental Biol Med. (2017) 39:1010428317705790. doi: 10.1177/1010428317705790 28635401

[40] Brufsky AM Dickler MN. Estrogen receptor-positive breast cancer: Exploiting signaling pathways implicated in endocrine resistance. Oncologist. (2018) 23:528–39. doi: 10.1634/theoncologist.2017-0423 PMC5947450 29352052

[41] Wong RSY. Apoptosis in cancer: From pathogenesis to treatment. J Exp Clin Cancer Res CR. (2011) 30:87. doi: 10.1186/1756-9966-30-87 21943236 PMC3197541

[42] Mohammad RM Muqbil I Lowe L Yedjou C Hsu H-Y Lin L-T. Broad targeting of resistance to apoptosis in cancer. Semin Cancer Biol. (2015) 35 Suppl:S78–S103. doi: 10.1016/j.semcancer.2015.03.001 25936818 PMC4720504

[43] Ouyang J Liu Z Yuan X Long C Chen X Wang Y. LncRNA PRNCR1 promotes breast cancer proliferation and inhibits apoptosis by modulating microRNA-377/CCND2/MEK/MAPK axis. Arch Med Res. (2021) 52:471–82. doi: 10.1016/j.arcmed.2021.01.007 33608112

[44] Levine B Kroemer G. Biological functions of autophagy genes: A disease perspective. Cell. (2019) 176:11–42. doi: 10.1016/j.cell.2018.09.048 30633901 PMC6347410

[45] Klionsky DJ Petroni G Amaravadi RK Baehrecke EH Ballabio A Boya P. Autophagy in major human diseases. EMBO J. (2021) 40:e108863. doi: 10.15252/embj.2021108863 34459017 PMC8488577

[46] Kalluri R LeBleu VS. The biology, function, and biomedical applications of exosomes. Science. (2020) 367:eaau6977. doi: 10.1126/science.aau6977 32029601 PMC7717626

[47] Zhang L Yu D. Exosomes in cancer development, metastasis, and immunity. Biochim Biophys Acta BBA - Rev Cancer. (2019) 1871:455–68. doi: 10.1016/j.bbcan.2019.04.004 PMC6542596 31047959

[48] Exosomes mediated transfer of lncRNA UCA1 results in increased tamoxifen resistance in breast cancer cells (n.). Available online at: https://pubmed.ncbi.nlm.nih.gov/27831634/ (Accessed November 30, 2023). 27831634

[49] Zhang Y Weinberg RA. Epithelial-to-mesenchymal transition in cancer: Complexity and opportunities. Front Med. (2018) 12:361–73. doi: 10.1007/s11684-018-0656-6 PMC6186394 30043221

[50] Shibue T Weinberg RA. EMT, CSCs, and drug resistance: The mechanistic link and clinical implications. Nat Rev Clin Oncol. (2017) 14:611–29. doi: 10.1038/nrclinonc.2017.44 PMC5720366 28397828

[51] Chen X Ding J-C Hu G-S Shu X-Y Liu Y Du J. Estrogen-induced LncRNA, LINC02568, promotes estrogen receptor-positive breast cancer development and drug resistance through both in trans and in cis mechanisms. Adv Sci Weinh. Baden-Wurtt. Ger. (2023) 10:e2206663. doi: 10.1002/advs.202206663 PMC10477896 37404090

[52] Early Breast Cancer Trialists’ Collaborative Group (EBCTCG) Davies C Godwin J Gray R Clarke M Cutter D. Relevance of breast cancer hormone receptors and other factors to the efficacy of adjuvant tamoxifen: Patient-level meta-analysis of randomised trials. Lancet Lond Engl. (2011) 378:771–84. doi: 10.1016/S0140-6736(11)60993-8 PMC3163848 21802721

[53] Goetz MP Bagegni NA Batist G Brufsky A Cristofanilli MA Damodaran S. Lasofoxifene versus fulvestrant for ER+/HER2- metastatic breast cancer with an ESR1 mutation: Results from the randomized, phase II ELAINE 1 trial. Ann Oncol Off J Eur Soc Med Oncol. (2023) 34:1141–51. doi: 10.1016/j.annonc.2023.09.3104 38072514

[54] Targeting and engineering long non-coding RNAs for cancer therapy | nature reviews genetics (n.). Available online at: http://www-nature-com-s.webvpn.njmu.edu.cn:8118/articles/s41576-024-00693-2 (Accessed May 10, 2024). 10.1038/s41576-024-00693-2 38424237

[55] Rinaldi C Mja W. Antisense oligonucleotides: The next frontier for treatment of neurological disorders. Nat Rev Neurol. (2018) 14(1):9–21. doi: 10.1038/nrneurol.2017.148 29192260

[56] Ransohoff JD Wei Y Khavari PA. The functions and unique features of long intergenic non-coding RNA. Nat Rev Mol Cell Biol. (2018) 19:143–57. doi: 10.1038/nrm.2017.104 PMC5889127 29138516

